# Arabidopsis TGA256 Transcription Factors Suppress Salicylic-Acid-Induced Sucrose Starvation

**DOI:** 10.3390/plants12183284

**Published:** 2023-09-16

**Authors:** Matthew E. Bergman, Sonia E. Evans, Xiahezi Kuai, Anya E. Franks, Charles Despres, Michael A. Phillips

**Affiliations:** 1 Department of Cell and Systems Biology, University of Toronto, Toronto, ON M5S 3G5, Canada; matthew.bergman@mail.utoronto.ca (M.E.B.); sonia.ehieromosele@mail.utoronto.ca (S.E.E.); anya.franks@mail.utoronto.ca (A.E.F.); 2 Department of Biological Sciences, Brock University, St. Catharines, ON L2S 3A1, Canadacdespres@brocku.ca (C.D.); 3 Department of Biology, University of Toronto Mississauga, Mississauga, ON L5L 1C6, Canada

**Keywords:** salicylic acid, sugar signaling, pathogen defense, NON-EXPRESSOR OF PATHOGENESIS RELATED GENES, metabolomics, 2*C*-methyl-D-erythritol-4-phosphate pathway

## Abstract

Salicylic acid (SA) is produced by plants in response to pathogen infection. SA binds the NONEXPRESSOR OF PATHOGENESIS-RELATED GENES (NPR) family of receptors to regulate both positive (NPR1) and negative (NPR3/4) plant immune responses by interacting with the clade II TGACG (TGA) motif-binding transcription factors (TGA2, TGA5, and TGA6). Here, we report that the principal metabolome-level response to SA treatment in Arabidopsis is a reduction in sucrose and other free sugars. We observed nearly identical effects in the *tga256* triple mutant, which lacks all clade II TGA transcription factors. The *tga256* mutant presents reduced leaf blade development and elongated hypocotyls, roots, and petioles consistent with sucrose starvation. No changes were detected in auxin levels, and mutant seedling growth could be restored to that of wild-type by sucrose supplementation. Although the retrograde signal 2-*C*-methyl-D-erythritol-2,4-cyclodiphosphate is known to stimulate SA biosynthesis and defense signaling, we detected no negative feedback by SA on this or any other intermediate of the 2-*C*-methyl-D-erythritol-4-phosphate pathway. Trehalose, a proxy for the sucrose regulator trehalose-6-phosphate (T6P), was highly reduced in *tga256*, suggesting that defense-related reductions in sugar availability may be controlled by changes in T6P levels. We conclude that the negative regulatory roles of TGA2/5/6 include maintaining sucrose levels in healthy plants. Disruption of TGA2/5/6-NPR3/4 inhibitory complexes by mutation or SA triggers sucrose reductions in Arabidopsis leaves, consistent with the ‘pathogen starvation’ hypothesis. These findings highlight sucrose availability as a mechanism by which TGA2/5/6 balance defense and development.

## 1. Introduction

**Figure 1:**
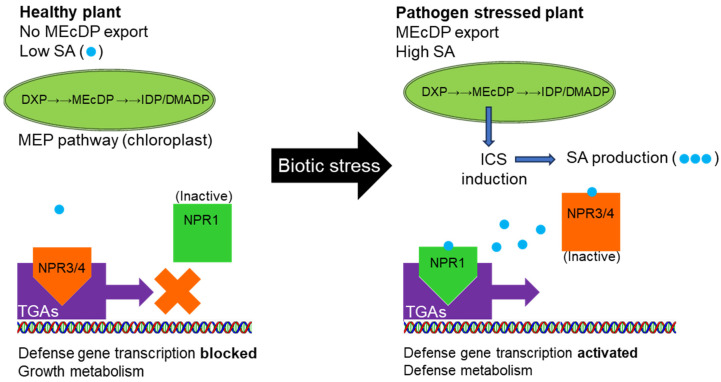
Schematic representing involvement of the isoprenoid intermediate 2C-methyl-D-erythritol-1,4-cyclodiphosphate (MEcDP) in retrograde signaling and activation of salicylic acid (SA) defenses. The SA receptors, NONEXPRESSOR OF PATHOGENESIS-RELATED GENES (NPR), regulate positive and negative functions of the TGACG motif-binding transcription factors (TGAs) according to the concentration of SA. At low SA concentrations, NPR3/4 bind TGA2/5/6 and repress expression of defense related genes. MEcDP activates SA production during plant immune responses to certain types of stress [9,10,11] which prompt export of MEcDP from the plastid and induction of ISOCHORISMATE SYNTHASE expression, which leads to SA accumulation. At high SA concentrations, NPR3/4 lose their inhibitory activity and SA-bound NPR1 binds TGAs, which stimulate defense gene expression.

Salicylic acid (SA) is a stress hormone deployed by plants in response to attack by biotrophic microbial pathogens [1] and phloem-sucking insect herbivores [2]. SA controls the expression of defense-related genes via two regulatory protein components. The first are SA receptors known as NONEXPRESSOR OF PATHOGENESIS-RELATED GENES (NPRs). In Arabidopsis, NPR1 is the most significant for defense signaling [3]. Upon SA binding, the complex relocates to the nucleus where it interacts with the second component, members of the TGACG (TGA) motif-binding transcription factors (TFs) [4], a family of basic leucine zipper (bZIP) proteins. The 10 TGA transcription factors in Arabidopsis are divided into five clades and control gene expression related to basal defense, systemic acquired resistance (SAR), and xenobiotic detoxification [5]. Clade II, consisting of TGA2, TGA5, and TGA6, play redundant roles in SAR where they induce expression of pathogen-related defense genes when activated by SA-bound NPR1 [6]. NPR3/NPR4, in contrast, repress transcription of these same genes in complex with TGA2/5/6 [7], and in their case, SA binding abolishes their inhibitory activity (Figure 1). Thus, while TGA2/5/6 positively activate defense signaling in response to SA accumulation, these same transcription factors exert negative feedback regulation over defense signaling when SA is absent [7,8]. In this way, small fluctuations in SA concentration simultaneously relieve inhibition and stimulate expression of hundreds of pathogenesis-related (PR) proteins, a process crucial to the SAR response [1].

To unravel the positive and negative regulatory properties of TGA256 within the SA signaling network, studies have relied heavily on the Arabidopsis *tga256* triple mutant defective in all three clade II TGA transcription factors [6]. Studies with this triple mutant line have established that, besides acting synergistically with WRKY transcription factors to induce expression of the key pathogen defense gene, PR1 [12], TGA2, TGA5, and TGA6 are also essential for UV-B-induced oxidative stress tolerance [13]. Despite their otherwise antagonistic regulatory effects, NPR1 and NPR4 coordinately amplify the SA signal, are required for pattern and effector triggered immunity (PTI and ETI), and regulate SA levels through modifications including glycosylation and 5-hydroxylation [14]. Although SA signaling generally inhibits jasmonic acid/ethylene (JA/ETH) induced defense responses, it may stimulate them to counteract necrotrophic pathogens [15]. MEcDP stimulates both SA and JA responsive genes and triggers accumulation of SA and the JA precursor 12-oxophytodienoic acid (but not JA itself) [16,17]. SA signaling is therefore complex and context-dependent, and although its main function relates to plant immunity against biotrophs, it helps coordinate responses to a variety of biotic and abiotic stressors in conjunction with NPRs and TGA transcription factors.

Because of the profound effects that SA exerts on the metabolic state of the plant, its biosynthesis is tightly controlled. SA biosynthesis is partly controlled by expression of the enzyme ISOCHORISMATE SYNTHASE (ICS), which catalyzes its biosynthesis from chorismate, the dominant of two biosynthetic routes [18]. The Arabidopsis genome encodes two ICS genes, and ICS1 is the isoform responsive to pathogen infection [19]. Its expression is under the control of the plant-specific transcription factors SARD1 and CBP60g [20], as well as additional regulatory proteins such as ENHANCED DISEASE SUSCEPTIBILITY 1 (EDS1) and DELLA [21]. However, the mechanisms by which immune receptors transduce their signal to them is far less understood.

There is some evidence that small molecule signaling may play a part in this signal transduction process. The 2-***C***-methyl-D-erythritol-4-phosphate pathway (MEP) supplies the universal intermediates isopentenyl and dimethylallyl diphosphate (IDP and DMADP) for isoprenoid biosynthesis in the chloroplast following condensation of pyruvate and glyceraldehyde 3-phosphate to 1-deoxy-D-xylulose 5-phosphate (DXP), the committed intermediate of the pathway in plants [22]. A metabolic intermediate in this route, 2-C-methyl-D-erythritol-2,4-cyclodiphosphate (MEcDP), appears to play a secondary role as a retrograde signal which promotes SA biosynthesis [23]. Under certain stress conditions, MEcDP leaves the chloroplast to serve this moonlighting function in defense signaling in the nucleus. In the course of this extraplastidic signaling role, MEcDP is further metabolized into free 2-*C*-methyl-D-erythritol (ME) and ME glucosides which alter its capacity to induce PR proteins [24]. MEcDP retrograde signaling has been implicated in the protein unfolding response [25], calcium signaling [26], and auxin-mediated growth [27] in addition to its role in activating SA-mediated plant defense. It is currently unknown if SA signaling impacts these MEP pathway derived signaling metabolites, for example, through negative feedback. Indeed, the impacts of SA on central plant metabolism in general remain poorly characterized.

Here, we sought to understand the effects of SA on central metabolism using the *tga256* mutant. We observed a morphological and metabolic phenotype in this mutant that suggests the negative (inhibitory) roles of TGA2/5/6 transcription factors are important to prevent SA-linked defense responses in the absence of pathogens. Therefore, *tga256* mutant plants unexpectedly display the metabolic signature of SA signaling due to loss of TGA inhibitory roles. These changes include major declines in free sugar availability. This adds to our understanding of clade II TGA function in the context of growth defense trade-offs and plant immunity.

## 2. Results

### 2.1. Clade II TGAs Maintain Sugar Homeostasis by Buffering SA-Induced Suppression of Central Metabolism

**Figure 2:**
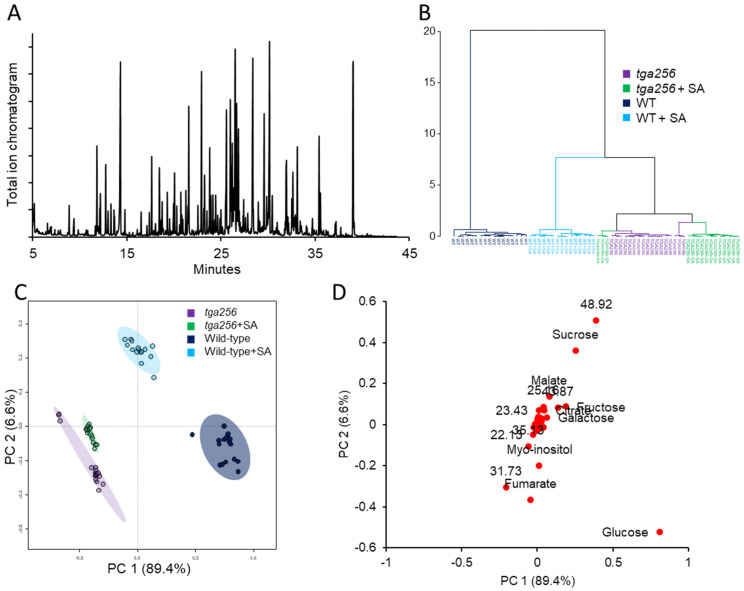
Untargeted metabolomics analysis of the *tga256* mutant by gas chromatography–mass spectrometry (GCMS). Polar metabolites from rosette tissue of the Arabidopsis *tga256* mutant or wild-type plants treated with salicylic acid (SA) or water (control) were extracted and derivatized for GCMS analysis (*n* = 5 plants per group). (**A**) Total ion chromatogram of a typical derivatized, polar metabolite analysis acquired in scan mode (*m/z* 50–550). A total of 166 features were identified per biological replicate and normalized to internal standards. (**B**) Hierarchical clustering analysis of groups showing Euclidean distance based on relative peak intensity of these features calculated using the Ward clustering algorithm. (**C**) Principal component analysis (PCA) score plot of these data show that 89.4% of the experimental variation is explained by component 1, compared to only 6.6% for component 2 on the vertical axis. (**D**) PCA loading plot of these data show that simple sugars account for most of the metabolite variation between groups.

Untargeted metabolome analysis of SA-induced wild-type and *tga256* mutant plants identified significant changes to primary metabolism linked to SA elicitation and clade II TGA transcription factors. Gas chromatography–mass spectrometry (GCMS) analysis of powdered leaf extracts yielded ~200 unique features representing derivatized, polar metabolites (principally organic acids, amino acids, and carbohydrates) (Figure 2A). A hierarchical clustering analysis of these four groups (wild-type or *tga256*, treated with SA or water (control)) showed a mostly group-specific separation into individual clades, although *tga256* controls and SA-treated plants did not cleanly segregate in all cases (Figure 2B). According to the nested structure of this clustering analysis, the tga256 mutant did not respond to SA treatment to the extent that wild-type plants did, but TGA-independent SA responses were nonetheless apparent. A principal component analysis (PCA) of these data confirmed these observations; namely, that most of the variation in the data set was observed between wild-type and *tga256* mutant plants, while a lesser but clearly observable subset of SA responses was apparent in the *tga256* mutant (Figure 2C). These three groups were distributed across the horizontal axis of the first principal component (PC1), which explained 89.4% of the variation. In contrast, the differences between SA-treated wild-type and control plants projected along the vertical axis (PC2) but accounted for only 6.6% of the variation (Figure 2C). A loadings plot of the variables from these PCA data comparing all four groups (Figure 2D) and a volcano plot directly comparing wild-type and *tga256* (Supporting Figure S1) confirmed that the most significant metabolic variables included a decrease in several carbohydrates in the *tga256* mutant line, specifically sucrose, glucose, fructose, and trehalose.

**Figure 3:**
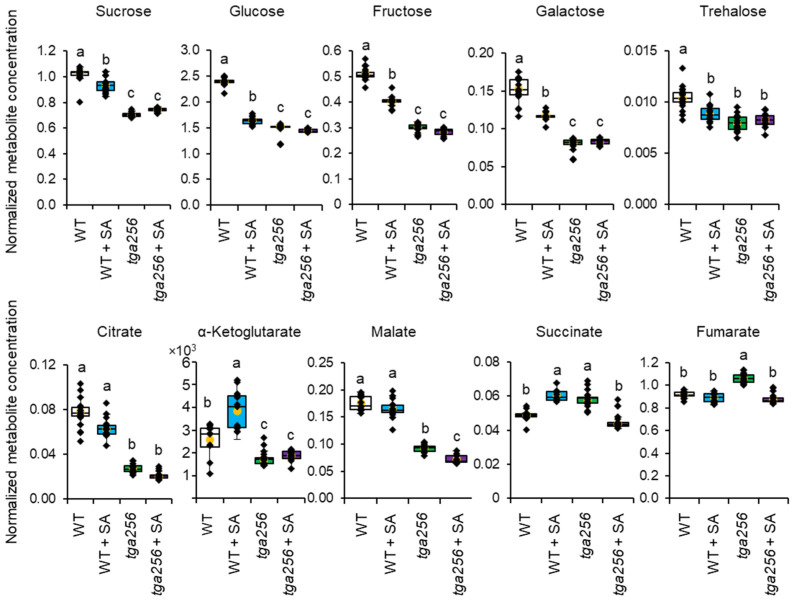
Selected metabolites from untargeted gas chromatography–mass spectrometry metabolomics analysis. The *y*-axis shows peak areas of analytes relative to internal standards, normalized to sample mass (mg dry weight) (see Section 4 for complete details). Yellow dots show average values, and individual plant values are shown as black dots (*n* = 15). The center line represents the median; upper and lower box limits indicate quartiles 1 and 3, respectively, and whiskers show the minima and maxima of the dataset. Groups with different letters have significantly different means (*p* ≤ 0.001) as calculated by a Tukey–Kramer test following a two-way ANOVA. Analyte peak areas were normalized to that of the internal standard (ribitol). WT, wild-type. SA, salicylic acid.

A univariate analysis that individually quantified changes in metabolites identified by PCA revealed that, while free sugars and the citric acid cycle intermediates citrate and malate declined in salicylic acid (SA)-treated wild-type plants, their declines in the *tga256* mutant were greater still. SA treatment of wild-type plants resulted in a 9% decrease in sucrose while the *tga256* mutant declined by ~32% with or without SA compared to wild-type controls (Figure 3). While sucrose constitutes the largest pool of carbohydrates, other free sugars such as glucose, fructose, and galactose showed a similar pattern on a normalized basis (Figure 3), as did trehalose, which is the breakdown product of the sucrose metabolism signal trehalose-6-phosphate (T6P) [28]. The TCA cycle intermediates citrate and malate also echoed this trend; SA provoked mild declines in their pool sizes in wild-type compared to untreated wild-type controls (10% for citrate and 24% for malate), but their decreases in the *tga256* mutant with (64% and 38%) or without SA induction (53% and 36%) were significantly larger compared to the same wild-type control plants. These relative magnitudes likely reflect the short-term effects of SA treatment versus the long term and developmental consequences of mutation at the *TGA2/5/6* loci. While our initial expectation was that *tga256* mutant plants would exhibit the opposite metabolic effects as SA elicitation due to loss of positive SA signaling, these results are in fact consistent with the observation that loss of clade II TGA negative regulatory activity exerts the most dominant influence on basal metabolism [8,29]. Hence, *tga256* mutants appear to have lost the ability to prevent suppression of central metabolism, their default role in healthy plants when SA levels are low, and the metabolic profile of the triple mutant resembles that of SA-elicited wild-type plants.

### 2.2. The MEP Pathway Is Not under Negative Feedback Regulation by SA

**Figure 4:**
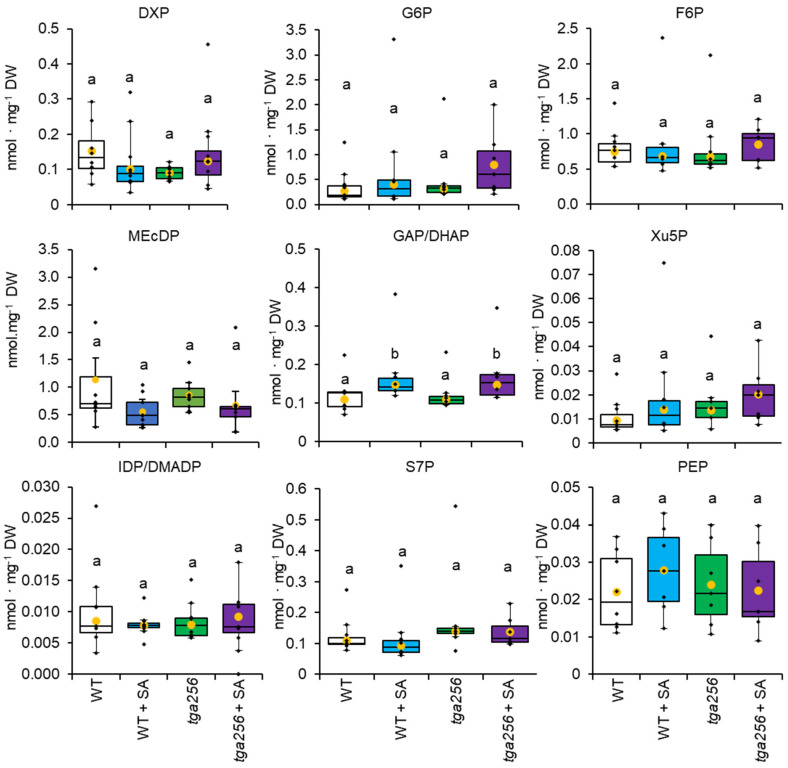
Targeted analysis of metabolites from central metabolism in wild-type and *tga256* mutant plants. Plants were screened for metabolic changes in the 2-*C*-methyl-D-erythritol-4-phosphate pathway or the Calvin-Benson cycle 24 h after spraying with salicylic acid (SA). Yellow dots show average values, and individual plant values are shown as black dots (*n* = 9). The center line represents the median; upper and lower box limits indicate quartiles 1 and 3, respectively, and whiskers show the minima and maxima of the dataset. DXP, 1-deoxy-D-xylulose-5-phosphate; MEcDP, 2-*C*-methylerythritol-2,4-cyclodiphosphate; IDP/DMADP, isopentenyl, and dimethylallyl diphosphate; G6P, glucose 6-phosphate; GAP/DHAP, glyceraldehyde 3-phosphate, and dihydroxyacetone phosphate (detected as a single peak); PEP, phosphoenolpyruvate; F6P, fructose 6-phosphate; sedoheptulose 7-phosphate; Xu5P, xylulose 5-phosphate; IDP and DMADP could not be resolved by this method. Groups with different letters have significantly different means (*p* ≤ 0.05) as calculated by a Tukey-Kramer test following a two-way ANOVA. Analyte peak areas were normalized to that of the internal standard. Quantification was based on an external standard curve corrected for recovery of the internal standard, 1-deoxyglucose 6-phosphate.

Targeted analysis of phosphorylated metabolites by liquid chromatography–tandem mass spectrometry (LCMS/MS) revealed no changes to intermediates of the MEP pathway and few differences to glycolytic or Calvin–Benson cycle intermediates. Compared to wild-type plants, *tga256* plants displayed no significant changes in concentration of DXP, MEcDP, IDP, or DMADP (Figure 4). Similarly, the concentrations of MEP pathway intermediates did not change in wild-type or mutant plants (Figure 4).

Intermediates of the Calvin–Benson cycle and glycolysis largely failed to respond to exogenous SA treatment with the exception of triose phosphate, which rose ~40% in both wild-type and *tga256* mutant plants following SA application (Figure 4). This result suggested that increases in glyceraldehyde 3-phosphate and dihydroxyacetone phosphate pools in response to SA were independent of TGAs. However, no changes in hexose phosphate pools were detected in this analysis (Figure 4). Other small differences in phosphorylated central metabolites between wild-type and mutant plants were not statistically significant (Figure 4), and SA treatment had no effect on their steady state levels. Overall, changes to central, phosphorylated metabolite pools were minimal in these study groups with the exception of triose phosphate, which changed equally in response to SA treatment independently of TGA activity.

### 2.3. The tga256 Mutant Displays Elongated Petioles, Hypocotyls, and Roots

**Figure 5:**
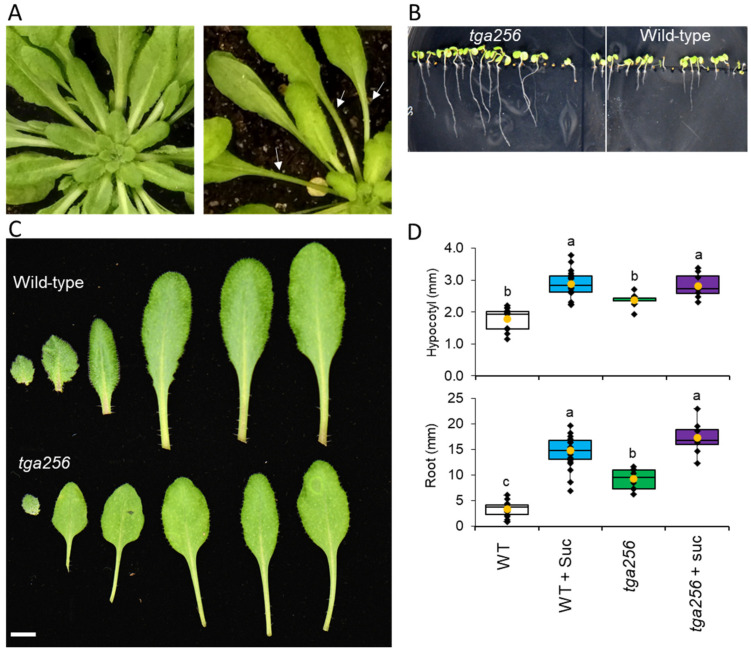
Morphological phenotype of the Arabidopsis *tga256* triple mutant. (**A**) Rosette of wild-type plants showing normal leaf blade and petiole morphology compared to the *tga256* mutant, which develops an elongated, spindly petiole (white arrows) with a reduced blade. (**B**) Root and hypocotyl growth assay showing accelerated root growth in the *tga256* mutant compared to wild-type on MS media. (**C**) Leaf series of wild-type and *tga256* mutant comparing petiole and blade morphology (bar = 5 mm). (**D**) Quantification of root and hypocotyl growth shown in C in wild-type and *tga256* mutant plants 10 days post germination on MS or MS + sucrose (suc) (1%) media. Yellow dots show average values, and individual plant values are shown as black dots (*n* = 9). The center line represents the median; upper and lower box limits indicate quartiles 1 and 3, respectively, and whiskers show the minima and maxima of the dataset. Groups with different letters have significantly different means (*p* ≤ 0.01) as calculated by a Tukey−Kramer test following a two−way ANOVA.

The decreased concentrations of key sugars in the *tga256* mutant led us to examine it for morphological changes that might explain its altered metabolic profile. Rosette stage, adult plants grown under short day (SD) conditions displayed developmental abnormalities in foliar morphogenesis (Figure 5A) compared to wild-type plants. When grown under identical conditions as wild-type plants (Supporting Figure S2), the *tga256* mutant exhibited a reduced leaf blade with an elongated, slender petiole (Figure 5A–C). Leaf blades of mutant plants were also less serrated than the wild-type and had a slight yellow pallor. The *tga256* mutant also exhibited elongated roots (Figure 5B). We therefore quantified root and hypocotyl lengths of wild-type and *tga256* plants with and without sucrose supplementation. Hypocotyls of *tga256* mutants were 33% longer than wild-type controls grown under the same conditions (Figure 5D). When the media was supplemented with sucrose, hypocotyls of both genotypes grew longer, and differences between groups disappeared (Figure 5D). This complementation suggests that the morphological phenotype we observed in *tga256* mutants was due to the lower sucrose concentrations we observed in untargeted metabolomics analysis (Figure 2 and Figure 3).

Root growth of *tga256* mutant plants displayed even larger differences than hypocotyls. Roots of *tga256* mutants were on average 172% longer than their wild-type counterparts (Figure 5D). When supplemented with sucrose, roots from both groups grew significantly longer than non-supplemented controls. As with hypocotyl growth, disappearances between mutant and wild-type root length disappeared on media supplemented with sucrose (Figure 5D).

### 2.4. The tga256 Triple Mutant Is Deficient in Gibberellin but Has Normal Auxin Levels

**Figure 6:**
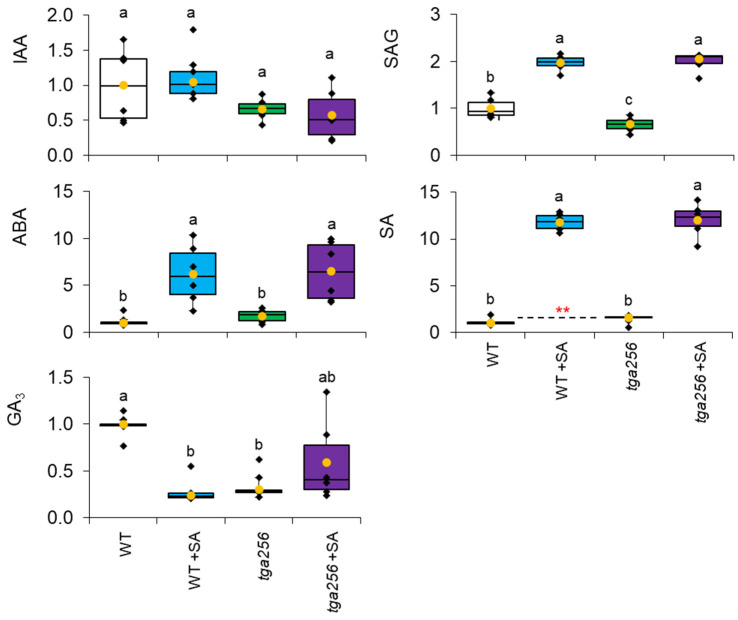
Relative phytohormone levels normalized to wild-type levels (WT). WT and *tga256* mutant plants treated with salicylic acid (SA) or water were analyzed using liquid chromatography–tandem mass spectrometry. Peak areas were normalized for sample weight and corrected to the area of internal standard tropic acid. Yellow dots show average values, and individual plant values are shown as black dots (*n* = 9). The center line represents the median; upper and lower box limits indicate quartiles 1 and 3, respectively, and whiskers show the minima and maxima of the dataset. Letters show significant differences between group means as calculated by a Tukey–Kramer test following a two-way ANOVA to test for equality of means (*p* < 0.05). IAA, indole acetic acid; ABA, abscisic acid; SAG, salicylic acid glucoside; SA, salicylic acid; GA3, gibberellic acid. Red asterisks indicate statistical significance based on a student’s *t*-test (** *p* < 0.01).

Based on the morphology of the *tga256* mutant, we examined auxin and other phytohormone concentrations to test for the involvement of growth regulators. Phytohormone profiling indicated there was no statistically significant difference in auxin (indole acetic acid; IAA) levels in the *tga256* mutant (Figure 6). Auxin levels were likewise unresponsive to exogenous SA treatment in either group. This observation indicated that the *tga256* phenotype was not the result of alterations to basal IAA levels. The other phytohormone implicated in stem elongation is gibberellin, which promotes cell elongation during skotomorphogenesis [30]. We anticipated gibberellin levels would be elevated in *tga256* if this phytohormone were responsible for its partially etiolated phenotype. However, *tga256* mutants and SA-treated wild-type plants had only ~30% of the gibberellin (GA_3_) content of wild-type (Figure 6; *p* < 10^−3^ in both cases). SA treatment of *tga256* plants restored GA_3_ to the wild-type control level (Figure 6). Mutant and wild-type plants were therefore sprayed with GA_3_ three times a week for three weeks to investigate the effects of complementing the gibberellin deficiency in *tga256*. However, GA3 treatment did not complement the *tga256* mutant leaf phenotype or otherwise affect leaf morphology compared to untreated wild-type or mutant plants (Supporting Figure S3).

Several other phytohormones also responded to SA treatment or demonstrated alterations in the *tga256* mutant line. Salicylic acid-2-O-β-D-glycoside (SAG) increased several fold in SA treated wild-type and *tga256* plants (Figure 6), verifying its uptake in these experiments and confirming its rapid conversion to its inactive, glycoside storage form [31]. Basal SA levels in *tga256* were ~58% higher than in wild-type plants. While this difference was significant in a direct comparison based on a student’s *t*-test (n = 5; *p* = 0.01), this effect fell below the significance threshold in an ANOVA which included SA-treated samples and greater variation. Abscisic acid (ABA) in both wild-type and *tga256* mutants responded to SA treatment with dramatic increases compared to their corresponding untreated controls (~6–6.5-fold increases, with *p* values < 0.01 for wild-type and *p* < 0.05 for the *tga256* mutant). Overall, these observations suggest that GA_3_ was the most affected phytohormone in *tga256*, and that none of the morphological or metabolic alterations was due to changes in auxin levels.

### 2.5. NPR3, NPR4, and PR1 Transcripts Are Responsive to SA Induction Independently of TGA256

**Figure 7:**
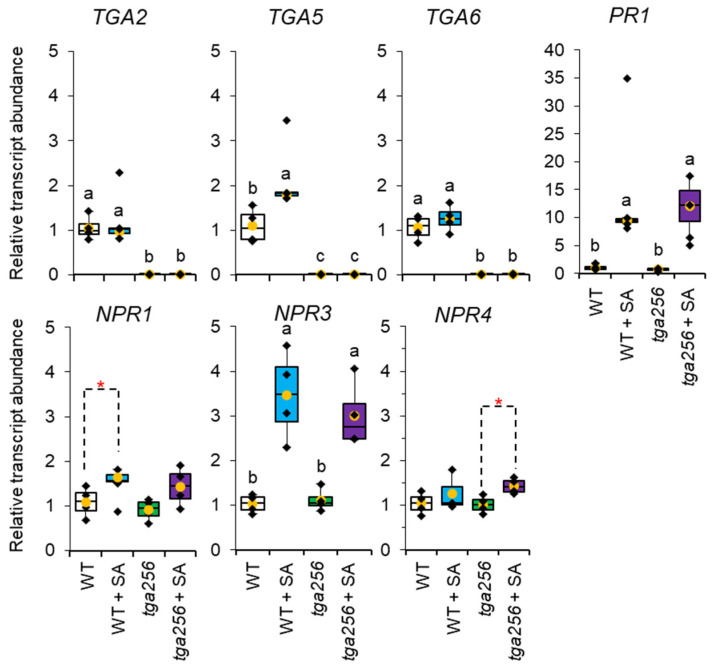
Response of *TGA*, *NPR*, and *PR* transcripts to salicylic acid induction in wild-type and *tga256* mutant plants. Relative transcript abundance was calculated using real-time quantitative PCR (*n* = 4). cDNA loading was corrected using the reference gene *RP2ls.* For box plots, the center line represents the median, upper and lower box limits indicate quartiles 1 and 3, respectively, and whiskers show the minima and maxima of the dataset. Yellow dots indicate the average of the data set. Groups with different letters have significantly different means (*p* ≤ 0.01) as calculated by a Tukey–Kramer test following a two-way ANOVA. Red asterisks indicate statistical significance based on a student’s *t*-test (* *p* < 0.05). WT, wild-type. SA, salicylic acid. Primer sequences are listed in supporting Table S3. cDNA sequences for primer design were downloaded from https://www.arabidopsis.org/ accessed on 11 April 2023.

Quantitative PCR (QPCR) assays confirmed the absence of *TGA2*, *TGA5*, and *TGA6* transcripts in the *tga256* mutant (Figure 7). *TGA5* expression was slightly induced by SA in wild-type plants. *NPR3* transcripts rose in response to SA treatment in wild-type (*p* < 0.001) and in the *tga256* mutant (*p* < 0.01), while the SA-induction of *NPR4* was only statistically significant in the mutant (*p* < 0.05). *NPR1* transcripts rose in the wild-type in response to SA treatment, consistent with previous reports which showed mild SA induction of NPR1 [32]. PR1 expression responded strongly to SA induction in wild-type and the *tga256* mutant (*p* < 0.01) (Figure 7). This was largely consistent with Fonseca and co-workers [8], although in our hands, *PR1* responsiveness to SA induction in *tga256* was comparable to that of wild-type plants. This distinction may be due to higher SA concentrations employed in our study (1 mM). With the exception of *TGA2/5/6*, the *tga256* mutant displayed nearly identical transcript levels to wild-type plants in both control and SA-treated plants.

## 3. Discussion

### 3.1. TGA2/5/6 Negative Regulatory Functions Are Required for Sugar Homeostasis

The *tga256* triple mutant was originally generated from a cross of two deletion mutants to investigate the role of clade II TGA transcription factors in SA-mediated plant immunity [6]. Since its deposition into public collections, it has become a widely used genetic resource to understand the positive and negative transcriptional regulation of pathogen responses orchestrated by clade II TGAs [7,8], the link between SA and ETH signaling [15,33,34], and the role of TGAs in tolerating UV and oxidative stress [13,35]. We selected it for metabolomic analyses to understand the effect of SA signaling on primary metabolism. The prominence of TGA2/5/6 in positive SA defense signaling led us to speculate that the triple mutant would be unable to transduce the same changes to central metabolism upon SA induction. However, we observed the opposite. Changes to sucrose and other free sugars were the main metabolic consequence of SA treatment, and this effect was even more exaggerated in the *tga256* mutant (Figure 2 and Figure 3). Evidently, the loss of the negative regulatory functions of TGA2/5/6 produces a more prominent effect on metabolism than loss of their positive roles, and the lifting of inhibitory effects of TGA2/5/6 on defense gene expression consequently exerts a stronger net effect on central metabolism. Negative gene regulation by TGA2/5/6 occurs in complex with NPR3 and NPR4 [7,36,37,38]. Among other effects, TGA2/5/6 negatively controls SA accumulation under stress conditions [8], and our observation that the *tga256* mutant accumulates higher levels of SA (Figure 6) is consistent with Fonseca et al. In light of this, it is unsurprising that loss of clade II TGAs produces a similar effect on the metabolome as SA treatment. These results indicate that, without the negative roles of TGA2/5/6-NPR3/4 to suppress SA defense signaling in healthy plants, the baseline metabolic state trends towards leaky SA induction, which is accompanied by declines in sucrose availability. This highlights the importance of TGA2/5/6 for maintaining carbohydrate pools in the disease-free state.

Declines in free sugars in plant cells in response to SA induction have been reported in other plant species. Exogenous SA treatment of narrow-leafed plantain (*Plantago lanceolata*) displayed reductions in sucrose, glucose, and fructose [39], and reductions in these same sugars were reported for ragwort (*Jacobaea vulgaris* and *Jacobaea aquatica*) [40]. In tobacco, SA treatment led to reductions in transcripts for sucrose metabolism [41]. The reduction in free sugars and photosynthesis may reflect an evolved strategy to limit sugar availability to pathogens during infection [42].

### 3.2. Altered Growth Patterns in tga256 Result from a Disruption in Sucrose Supply

Despite the variety of detailed molecular investigations reported with this triple mutant, its morphological phenotype has not previously been noted. We considered how the observed perturbations to central metabolism might result in the *tga256* morphological phenotype. Its partially etiolated appearance bears some similarities to the shade avoidance phenotype, which consists of elongated hypocotyls and petioles, reduced blade expansion, and epinastic growth in response to low R:FR light ratios [43,44]. Shade avoidance responses are orchestrated through increases in auxin biosynthesis [45,46], and the absence of changes in auxin level in *tga256* (Figure 6) led us to rule this out as a likely explanation. Moreover, *tga256* levels of gibberellin, which promote etiolated growth and increased hypocotyl elongation [47], were lower, rather than higher, compared to the wild type.

Sugar signaling may provide a simpler explanation for the observed growth inhibition in *tga256*. Transport of sucrose from source to sink is a major determinant of plant growth [48]. Sucrose, along with glucose and T6P, can regulate growth and metabolic processes independently of basal functions [49]. This is consistent with the complementation of the growth phenotype of the *tga256* mutant when grown on sucrose media (Figure 5). Sucrose limitation, independent of auxin changes, inhibits axillary bud growth in peas [50], a finding which challenged long-held notions of apical dominance. Sucrose acts as a signal through sugar-sensing proteins and controls many morphological, physiological, and hormonal processes in plants [51,52,53]. Sucrose is also an important determinant of pathogen resistance [54,55].

The Arabidopsis proteins which carry out efflux of sucrose from the cytosol into the phloem are SUGARS WILL EVENTUALLY BE EXPORTED TRANSPORTERS (SWEETs) [56,57]. SWEET proteins are involved in pathogen resistance genes [58] and have been identified as susceptibility (S) genes [59]. Many if not most pathogens induce host SWEET transporters to gain access to the plant sugar resources for nourishment [60]. The precise role for SWEET transporters during plant–pathogen interactions has evaded simple definition, and two non-exclusive hypotheses exist to explain plant metabolic responses to biotrophic pathogen infection: ‘sugar signaling’ and ‘pathogen starvation’ [55]. Our results highlight the starvation hypothesis, based on reductions in sucrose in the *tga256* mutant with leaky SA induction and in SA-treated wild-type plants. The long-term developmental consequences of sucrose deficiency in *tga256* provide the most likely explanation for the developmental phenotype we describe here.

### 3.3. Role of Small Metabolite Signals in Clade II TGA Defense Signaling

Small metabolite signals play important roles in defense signaling. MEcDP accumulates in response to a variety of stressors including high light [9] and herbivory [10], but was originally noted for its role in the resistance to biotrophic pathogens [11]. Its export from the plastid [61] during stress leads to the activation of SA signaling [23,24], but it also participates in crosstalk between SA and jasmonate signaling pathways [17]. We investigated potential feedback of SA or TGA2/5/6 transcriptional regulation on MEcDP levels but observed no direct effect of either in wild-type plants (Figure 4). We furthermore did not observe changes in DXP, the committed intermediate of the MEP pathway, or in IDP or DMADP, the pathway’s end products. These observations lead us to conclude that, while MEcDP acts as a retrograde signal which stimulates SA biosynthesis and defense signaling, there is no readily detectable negative feedback regulation of SA on MEcDP signal strength. Follow up studies will investigate the influence of TGA2/5/6 transcriptional regulation on retrograde signal activity.

Another small metabolite signal with relevance to our results is T6P, a homeostatic regulator of sucrose levels in plants [62]. T6P determines how much sucrose is synthesized, and when the plant initiates developmental programs which increase sucrose demand. T6P also controls the release of bud dormancy via sucrose availability [63]. Alterations in T6P metabolism which raise or lower T6P activity provoke corresponding changes in leaf, petiole, and root elongation phenotypes in Arabidopsis [64] with interesting parallels to the morphological phenotype we describe here for *tga256*. The *tga256* mutant displayed significantly reduced levels of the T6P breakdown product, trehalose (Figure 3), a reliable indicator of T6P levels [65]. SA was previously shown to induce expression of a specific isoform of T6P synthase (TPS) in poplar [66], and SA-responsive cis-acting elements were detected in the promoter regions of TPS genes in cucumber (*Cucumis sativus* L.) [67] and sweet orange (*Citrus sinensis*) [68]. Our evidence implicates T6P signaling in TGA-mediated growth and immunity trade-offs. Future studies will examine the impact of clade II TGA transcriptional regulation on T6P activity and sucrose availability in the context of pathogen defense.

## 4. Materials and Methods

### 4.1. Plant Growth and Treatments

Arabidopsis thaliana wild-type (ecotype Columbia 0) and *tga256* mutant plants were grown in soil as previously described [69]. The *tga256* triple mutant line was described previously [6]. For metabolite extraction, wild-type and mutant plants were grown under SD conditions (8/16 h light/dark, 21 °C) for 6 weeks and watered three times weekly. Plants were grown individually in 8.5 cm square pots. For petiole measurements, genotypes were arranged in an alternating pattern within each tray to ensure identical watering, fertilizer, and light (Supporting Figure S2), and blade surface area and petiolar length were photographed using a Nikon D7500. Exogenous SA was applied by spraying at either 400 μM for 4 h (untargeted metabolome analysis) or 1 mM for 24 h (all other treatments) prior to liquid nitrogen freezing, and controls sprayed with water only were performed in parallel. Following freezing, plants were ground to a fine powder in a mortar and pestle and lyophilized to dryness against a vacuum of 25 μbar for 48 h. Lyophilized tissue samples were stored at −80 °C prior to metabolite extraction. For gibberellin treatments, 4-week-old *tga256* mutant and wild-type plants were sprayed with 50 μM exogenous GA_3_ (or water) 3 times a week for 3 weeks and then photographed. Petioles, leaf blades, hypocotyls, and roots of mutant and wild-type plants were photographed with a Nikon D7500 digital camera.

### 4.2. Measurement of Hypocotyl Length

Hypocotyls were measured in 10-day-old seedlings grown on sterile plates containing 5 mM MES buffer (pH 6.0), 3.225 g∙L-1 MS basal salts, and 4.5 g∙L^−1^ phytagel. Where noted, plates were supplemented with 1% (*w*/*v*) sucrose. Plates were stratified for 5 days at 4 °C before transfer to long day (LD, 16/8 h light/dark, 24 °C) conditions with 80 μmol∙m^−2^∙s^−1^.

### 4.3. GCMS Analysis

Unless otherwise noted, all standards and reagents were purchased from Sigma-Millipore, including high-purity solvents. Untargeted GCMS analysis of all plant samples was carried out as described [24,70], with minor exceptions. Briefly, 5 mg lyophilized tissue was extracted once with CHCl3, followed by two extractions with 70% methanol containing 5 μg ribitol as an internal standard. After pooling and centrifuging the extracts, the supernatant was dried under a nitrogen stream and resuspended in pyridine with methoxylamine for methyoximation of ketones and aldehyde groups, followed by trimethylsilylation just prior to injection on an Agilent Technologies 7890B GC coupled to a 5977B mass selective detector. The stationary phase was an HP5-ms capillary column (30 m × 0.25 mm i.d., 0.25 μm film thickness; Agilent Technologies) with helium as carrier gas at a constant flow of 1.0 mL∙min^−1^ and split injection mode (1:10 split ratio) with the injection port at 250 °C. The column oven temperature linearly increased from 70 to 325 °C at a rate of 5 °C∙min^−1^. Ionization was performed using electron impact in positive mode (+70 eV), and the analyzer acquired mass data in scan mode (*m/z* 50–550) with a scan rate of 2.9 Hz. Peak integration was performed with the Agile 2 integrator of MassHunter Qualitative Analysis (Agilent Technologies, Santa Clara, California, USA, version 10.0). Approximately 200 consistent features were identified across samples representing highly abundant polar central metabolites. Aligned peak tables were normalized to the internal standard (ribitol) peak area and sample mass for statistical analysis. Peak annotation relied on a combination of authentic standards, the NIST14 database, and the Golm Metabolome Database [71]. Statistical analysis and identification of significant features was carried out using Metaboanalyst [72].

### 4.4. LC-MS/MS Analysis

Highly polar phosphorylated metabolites were extracted from lyophilized plant tissue as described [69]. This was based on a previously described protocol [24], except that all components were maintained at or below 4 °C at all times, and the internal standard was 2-deoxy-D-glucose-6-phosphate. Briefly, a 10 mg tissue aliquot was vortexed with 250 μL ice-cold 50% (*v*/*v*) acetonitrile containing 10 mM ammonium acetate (pH 9.0) for 20 min. After cold centrifugation at 16,000× *g* for 10 min, the supernatant was transferred to a fresh tube and pooled following a second extraction with 50% (*v/v*) acetonitrile with 10 mM ammonium acetate. The extracts were lyophilized overnight, resuspended in ice-cold 10 mM ammonium acetate pH 9 (100 μL) and back-extracted with 100 μL chloroform. Phases were separated via centrifugation, and the upper, aqueous phase was diluted with 1 vol acetonitrile and filtered prior to analysis.

Targeted analysis of phosphorylated central metabolites was carried out using an Agilent 1290 series II ultrahigh pressure liquid chromatograph (LC) coupled to a Sciex 4500Qtrap triple quadrupole mass spectrometer. Chromatographic separation was performed using an XBridge BEH amide hydrophilic interaction chromatography column (2.1 mm × 150 mm, 2.5 μm particle size; Waters Corporation, Milford, Massachusetts, USA) with solvent gradient A (Supporting Table S1). Mass spectrometry parameters (mass transitions, voltage settings, and electrospray ion source settings) used during data acquisition appear in Supporting Table S2. Q1 and Q3 operated at unit resolution, and each transition was allocated a dwell time of 50 ms. The instrument was controlled with Analyst software version 1.7.2, and data analysis was performed with Sciex OS version 2.0.0. For quantification, peak areas were normalized to the internal standard and compared to the linear regression obtained from the calibration curve.

Phytohormones were extracted from 10 mg lyophilized tissue using 1 mL methanol containing 2 ng tropic acid by vortexing for 15 min. The extract was centrifuged at 16,000× *g* for 10 min, the supernatant transferred, and the pellet re-extracted with methanol (1 mL) in the same way. The supernatants were pooled and dried under vacuum and resuspended in methanol (100 μL). These methanol extracts were analyzed on the same instrument described above but using a Zorbax Eclipse XDB-C18 RRHT chromatography column (4.6 mm × 50 mm, 1.8 μm particle size; Agilent Technologies) and gradient B (Supporting Table S1). Statistical analysis was carried out using outlier-trimmed data, where outliers were determined as >1.5 times the interquartile range outside of quartiles one and three, and statistical significance was determined using a two way ANOVA and Tukey-Kramer comparison of means except where otherwise noted. Relative concentrations are based on the mean of wild-type controls unless otherwise stated.

### 4.5. Transcript Profiling

Total RNA was extracted from ground Arabidopsis rosette tissue using the Maxwell RSC Plant RNA kit (Promega) and quantified using a DS-11 Spectrophotometer (DeNovix^®,^ Wilmington, DE, USA). cDNA was reverse-transcribed using the Superscript IV Reverse Transcriptase kit (Invitrogen) according to manufacturer’s instructions and diluted 1:10 with pure water before use. Quantitative PCR assays were performed in a Bio-Rad CFX96 Touch Real-Time PCR Detection System with a reaction volume of 10 μL. Supporting Table S3 lists primer sequences for genes of interest and reference genes used in this study. Normalization using reference genes and calculation of fold change were performed as described [73].

## 5. Conclusions

When SA defense gene expression cannot be adequately repressed in healthy Arabidopsis plants, for example, when the relevant TGA transcription factors are mutated, leaky SA signaling trends toward defensive metabolism, even though no pathogens are present. We observed that one of the consequences of SA induction (constitutively in mutant plants or through application of exogenous SA) is a decline in available sugars, possibly to limit carbohydrate resources to invading pathogens, consistent with the so-called ‘pathogen starvation’ hypothesis [55]. Our study suggests that TGAs prevent these drastic metabolic responses except when serious infection compels it. Chronic effects of mutant plants developing in a perpetual state of low-level SA induction are likely responsible for the phenotype we observe in *tga256*, which includes reduced blade development and elongated hypocotyl and root growth. These results add to our understanding of carbohydrate dynamics as a mechanism by which plants balance growth and defense.

## Data Availability

All data pertaining to this manuscript are included in the text and supplementary materials.

## Supplementary Materials

The following supporting information can be downloaded at: https://www.mdpi.com/article/10.3390/plants12183284/s1, Figure S1: Volcano plot of significant metabolic features in the *tga256* mutant line. Figure S2: Cultivation of wild-type and *tga256* mutant plants for phenotypic comparison. Figure S3: Leaf series following gibberellin application to *tga256* mutant plants. Table S1: HPLC gradients for separation of phosphorylated metabolites and phytohormones. Table S2: MS/MS parameters for analysis of phosphorylated metabolites and phytohormones. Table S3: MS/MS parameters for analysis of phosphorylated metabolites and phytohormones.
