# Supplementary material for: Arabidopsis TGA256 Transcription Factors Suppress Salicylic-Acid-Induced Sucrose Starvation
Source: Plants (Basel). 2023 Sep 16;12(18):3284. doi: 10.3390/plants12183284 (PMC10534317; doi:10.3390/plants12183284)
Supplement: Supplementary file 1 [file plants-12-03284-s001.zip › plants-2596660-supplementary.pdf]

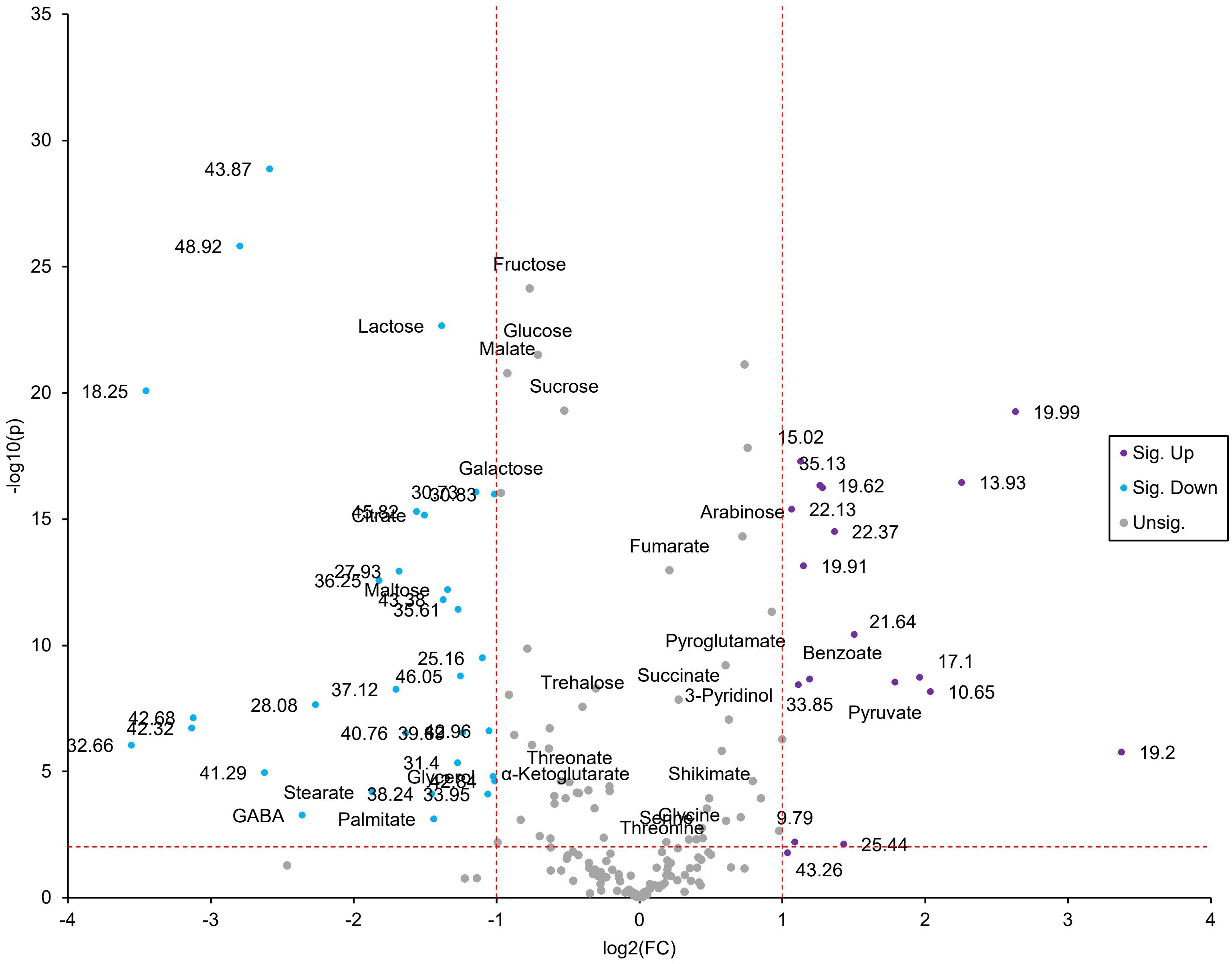

Supporting figure S1. Volcano plot of significant metabolic features in the *tga256* mutant line. Up (purple) and down regulated features (blue) were identified by untargeted gas chromatography – mass spectrometry (GCMS) metabolomics. Fold change in abundance of each analyte was calculated relative to wild-type plants (n = 6). Significant features identified by this analysis are those with a $-\log_{10}P$ value > 2 and a $\log_2$ fold change (FC) value >1 (sig. up) or <-1 (sig. down), as indicated by the dashed red lines. Features without definitive identification are marked with retention time (RT). Normalization of each metabolic feature was accomplished by converting to a fraction of to the internal standard peak area (D-ribitol). In this analysis, all features are equally weighted regardless of molar concentration. Unidentified metabolites labeled with RT are generally low concentration metabolites which could not be annotated.

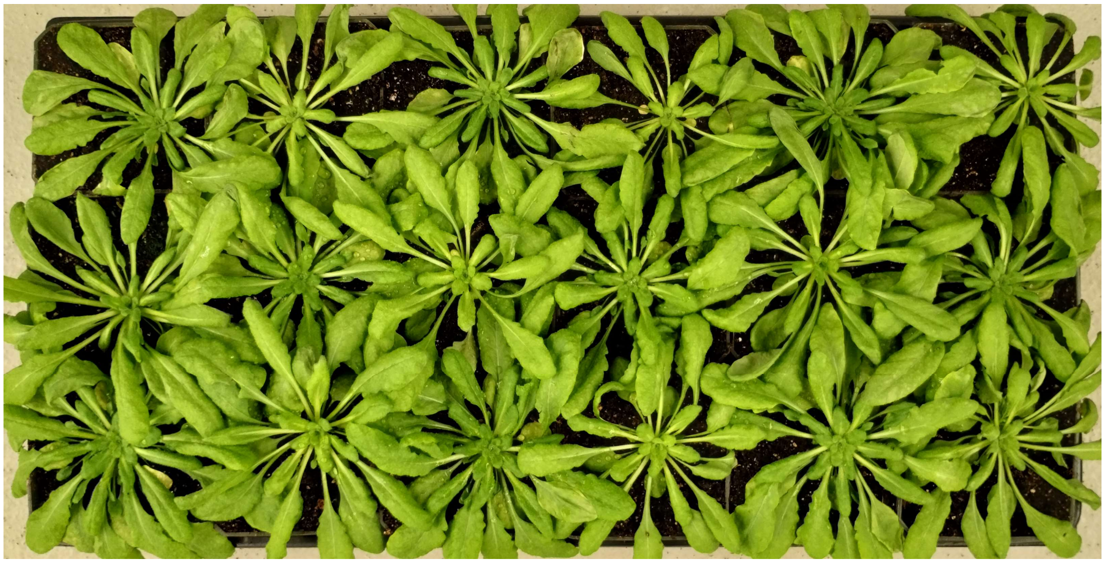

Supporting figure S2. Cultivation of wild-type and *tga256* mutant plants for phenotypic comparison. Wild-type and mutant plants were grown in an alternating pattern with equal watering, fertilizer and irradiance to minimize environmental effects. Plants were flash frozen in liquid nitrogen to harvest for metabolite analysis. Plant IDs (left to right, rows numbered from top to bottom) are as follows: Row 1: WT-1, tga256-1, WT-2, tga256-2, WT-3, tga256-3; Row 2: tga256-4, WT-4, tga256-5, WT-5, tga256-6, WT-6; Row 3: WT-7, tga256-7, WT-8, tga256-8, WT-9, tga256-9.

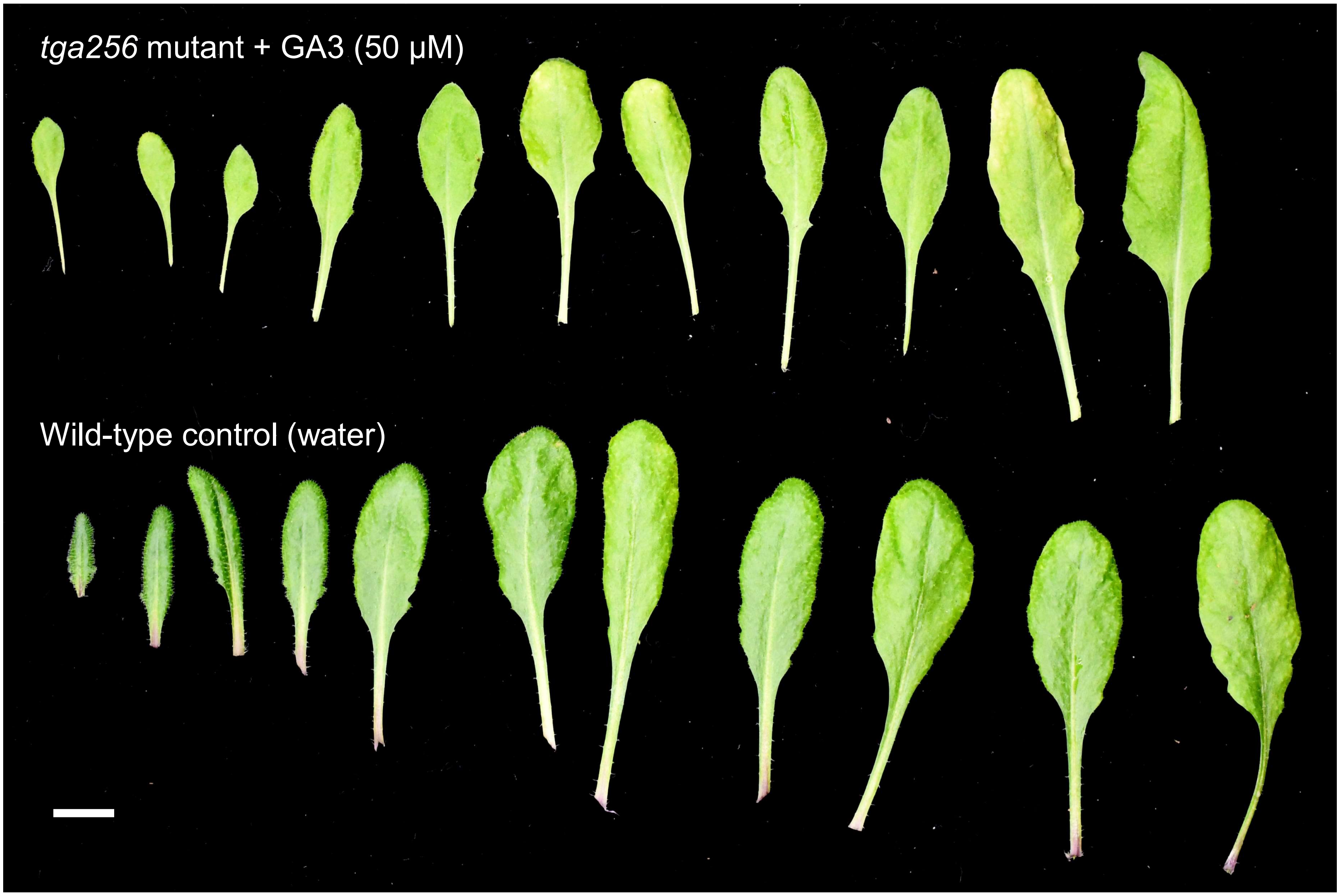

Supporting figure S3. Leaf series following gibberellin application to *tga256* mutant plants. Gibberellin levels in mutant plants were only ~33% of wild-type levels, and mutants were sprayed with exogenous 50 µM GA3 3 times a week for 3 weeks to determine whether gibberellin supplementation could restore wild-type leaf morphology. Following 3 weeks of GA3 treatment, there was no observable complementation of mutant leaf or petiole phenotype.

Supporting Table S1. HPLC gradients for separation of phosphorylated metabolites and phytohormones.

Gradient	Time (min)	Solvent A (%)	Solvent B (%)
Gradient A[a]	0.5 mL/min	20 mM NH_4HCO_3 pH 10.5	80% acetonitrile
	0	0	100
	5	16	84
	10	16	84
	11	40	60
	15	40	60
	15.1	0	100
	30	0	100
Gradient B[b]	1.1 mL/min	0.1% formic acid	Acetonitrile
	0	95	5
	0.5	95	5
	9.5	42	58
	9.51	0	100
	11	0	100
	11.01	95	5
	14	95	5

[a] Gradient A was performed on an XBridge BEH amide hydrophilic interaction chromatography column (2.1 × 150 mm, 2.5 µm particle size; Waters Corporation)

[b] Gradient B was performed on a Zorbax Eclipse XDB-C18 RRHT chromatography column (4.6 × 50 mm, 1.8 µm particle size; Agilent Technologies)

Supporting Table S2. MS/MS parameters for analysis of phosphorylated metabolites and phytohormones.

ID	Gradient	Mode	Q1 (m/z)	Q3 (m/z)	Dwell time (s)	DP	EP	CE	CXP	CUR	CAD	IS	TEM	GS1	GS2
MEcDP	A	(-)	277	79	50	-30	-10	-65	-11	20	10	-4000	500	60	30
IDP/DMADP	A	(-)	245	79	50	-45	-6	-24	-6	20	10	-4000	500	60	30
DXP	A	(-)	213	79	50	-30	-10	-40	-8	20	10	-4000	500	60	30
2-DGP	A	(-)	243	79	50	-35	-10	-62	-7	20	10	-4000	500	60	30
PEP	A	(-)	167	79	50	-5	-10	-14	-5	20	10	-4500	700	60	30
GAP	A	(-)	169	97	50	-35	-10	-20	-20	20	10	-4500	700	60	30
2-DGP	A	(-)	243	79	50	-35	-10	-62	-7	20	10	-4500	700	60	30
DHAP	A	(-)	169	97	50	-23	-10	-15	-7	20	10	-4500	700	60	30
Xu5P	A	(-)	229	97	50	-5	-10	-18	-6	20	10	-4500	700	60	30
G6P/F6P	A	(-)	259	97	50	-17	-8	-19	-9	20	10	-4500	700	60	30
S7P	A	(-)	289	97	50	-38	-5	-22	-7	20	10	-4500	700	60	30
IAA	B	(-)	174	130	10	-25	-9	-14	-2	25	7	-4500	650	60	60
SA	B	(-)	137	93	10	-20	-8	-24	0	25	7	-4500	650	60	60
SAG	B	(-)	299	137	10	-20	-10	-18	-2	25	7	-4500	650	60	60
ABA	B	(-)	263	153	10	-35	-12	-22	-2	25	7	-4500	650	60	60
Tropate	B	(-)	165	103	5	-40	-9	-14	-6	25	7	-4500	650	60	60
IAA	B	(+)	176	130	50	40	10	20	10	25	10	4500	650	60	60
GA_3	B	(-)	345	239	10	-79	-10	-32	-10	25	7	-4500	650	60	60

DP, declustering potential; EP, entrance potential; CE, collision energy, CXP; cell exit potential; Q1, quadrupole 1 mass; Q3, quadrupole 3 mass; CUR, curtain gas; CAD, collision gas; IS, ionspray voltage; GS1, ion source gas 1; GS2, ion source gas 2; MEcDP, 2*C*-methyl-D-erythritol 2,4-cyclodiphosphate; IDP, isopentenyl diphosphate; DMADP, dimethylallyl diphosphate; DXP, 1-deoxy-D-xylulose 5-phosphate; 2-DGP, 2-deoxy-D-glucose 6-phosphate; PEP, 2-phosphoenolpyruvate; GAP, D-glyceraldehyde 3-phosphate; G3P, DHAP, dihydroxyacetone phosphate; Xu5P, xylulose 5-phosphate; S7P, sedoheptulose 7-phosphate; IAA, indole-3-acetic acid; SA, salicylic acid; SAG, salicylic acid-2-O-β-D-glycoside; ABA, abscisic acid, GA_3, gibberellic acid.

Supporting Table S3. Primer sequences for quantitative PCR assays.

Primer name	Sequence (5'→3')
TGA2-F	GAGACAGTTGATGGGCATAAATAACC
TGA2-R	TTAGAGCTCGTAGCCGTGAGAAG
TGA5-F	ACAACAATCGCTAGACATAAATAACT
TGA5-R	TAAGAGCACGTAACCGCAATGTA
TGA6-F	ACGACAGGTAATGGGCATCAATAGCT
TGA6-R	TAAGAGCACGTAATCGAGATGAA
NPR1-F	TTGCCGGAAGAGCTTGTTAAAG
NPR1-R	GAGTCAAGTGCCTTATGTACATTCGA
NPR3-F	AGTTCCTCCCGAAGTAGCAGAGA
NPR3-R	TGAGAATTTTACCGATTCTTTCAAGC
NPR4-F	GGAGCTTCCTTTAGAAGTATTGGAAAA
NPR4-R	TGAGTACTTTCCCTGTTCTCTCTATC
PR1-F	GCAGCCTATGCTCGGAGCTA
PR1-R	AGACGCCAGACAAGTCACCG
RP2lsF	GAAGGCAAAGGAAGGCAGAATCAG
RP2lsR	GCAATACTCCACGGAACACCAAG
APT1F	GTTGCAGGTGTTGAAGCTAGAGGT
APT1R	TGGCACCAATAGCCAACGCAATAG
